# New York Medical Intelligencer

**Published:** 1845-12

**Authors:** 


### NEW YORK MEDICAL INTELLIGENCER.

This is the title of another periodical devoted to medicine, and emanating from the same city, which has lately taken its place among the journals of the day. The seventh number has been placed on our table. It is in the form of a newspaper, well printed, and contains 16 pages; thirteen numbers will constitute a volume, and the subscription is $2 per annum. It is edited by *D. S. Meikleham*, M.D. Like its young contemporary above noticed, it has a full table of contents, which is a feature that will command the applause of every reader, and especially of every editor. Another feature that we like in it, is its excellent arrangement. The matter is arranged under distinct heads, by which the reader may refer without trouble to any topic concerning which he is in search of instruction. It purports to be eclectic, and this number consists entirely of selected articles. They are also select, evincing in the editor capacity for the task he has assumed. We feel assured, from the specimen before us, that the Intelligencer will be found to be an interesting and valuable medical newspaper. Y.

